# Route-specific adverse effects of ondansetron: an overlooked concern in Asian clinical practice

**DOI:** 10.1097/MS9.0000000000005134

**Published:** 2026-05-15

**Authors:** Muhammad Ubaid Ur Rehman, Noor Un Nisa, Rafiullah Hotak

**Affiliations:** a Gujranwala Medical College, Gujranwala, Pakistan; b Jinnah Sindh Medical University, Karachi, Pakistan; c Faculty of Medicine, Nangarhar University, Jalalabad, Afghanistan

*To the Editor*,

Ondansetron, a 5-HT_3_ receptor antagonist, is among the most commonly prescribed antiemetics in routine clinical practice. Its effectiveness in perioperative care and chemotherapy-induced nausea and vomiting is well established. However, route-specific adverse effects remain under-recognized in local prescribing patterns. Intravenous (IV) administration, particularly when given as a rapid bolus, has been associated with Q-T interval prolongation, hypotension, and bradyarrhythmias, whereas oral use more frequently results in constipation and headache. Despite international safety warnings, awareness of these differential risks appears limited in Pakistan.

According to the U.S. Food and Drug Administration’s safety communications, rapid IV administration or higher doses of ondansetron increase the risk of QT prolongation and potentially serious arrhythmias^[^1,2^]^. These concerns are further supported by a recent meta-analysis demonstrating QTc prolongation across both oral and IV routes, with elderly patients and those with comorbidities being particularly vulnerable[3].

Case reports have also described torsades de pointes and clinically significant bradyarrhythmias following therapeutic doses of ondansetron[4]. Constipation associated with oral administration has been reported in nearly 9% of patients in randomized trials[5]; however, this estimate is derived from studies involving patients with irritable bowel syndrome with diarrhea (IBS-D), which may limit generalizability to broader clinical use. Local evidence remains sparse. A 2024 report published in the *Journal of the Pakistan Medical Association* documented IV ondansetron-induced QT prolongation and highlighted limited clinician awareness, as well as the absence of routine monitoring protocols.[6] Pharmacovigilance systems remain underdeveloped, and adverse drug reactions are infrequently reported. Furthermore, IV ondansetron is often preferred even for mild symptoms, although oral therapy may offer a safer and equally effective alternative in many cases.

Clinical risks are further amplified in patients with baseline QT prolongation, structural heart disease, hepatic impairment, or electrolyte disturbances. Rare but serious outcomes have been reported, including a case of asystole requiring resuscitation after repeated IV dosing, underscoring the importance of cautious use and appropriate monitoring.[7]

We suggest that Pakistani clinical practice align more closely with established safety recommendations. First, oral administration should be preferred when clinically appropriate. Second, IV ondansetron should be administered at recommended slow infusion rates rather than rapid bolus injections. Third, ECG monitoring should be considered for high-risk patients. Finally, strengthening pharmacovigilance systems and improving prescriber education could help reduce preventable adverse events.

Although route-specific adverse effects of ondansetron are well-recognized internationally, they remain underreported in Pakistan. Greater awareness, rational route selection, and adherence to safer prescribing practices may help prevent avoidable morbidity and improve patient safety.

## Data Availability

Not applicable.
